# Effect of hyperbilirubinemia on the accuracy of continuous non-invasive hemoglobin measurements in liver transplantation recipients

**DOI:** 10.1038/s41598-024-55837-5

**Published:** 2024-03-01

**Authors:** Soo Bin Yoon, Chul-Woo Jung, Taeyup Kim, Hyung-Chul Lee

**Affiliations:** Department of Anesthesiology and Pain Medicine, Seoul National University College of Medicine, Seoul National University Hospital, Seoul, Republic of Korea

**Keywords:** Laboratory techniques and procedures, Anaemia, Sensors and probes

## Abstract

This study evaluated the effect of hyperbilirubinemia on the accuracy of continuous non-invasive hemoglobin (SpHb) measurements in liver transplantation recipients. Overall, 1465 SpHb and laboratory hemoglobin (Hb) measurement pairs (n = 296 patients) were analyzed. Patients were grouped into normal (< 1.2 mg/dL), mild-to-moderate (1.2–3.0 mg/dL), and severe (> 3.0 mg/dL) hyperbilirubinemia groups based on the preoperative serum total bilirubin levels. Bland–Altman analysis showed a bias of 0.20 (95% limit of agreement, LoA: − 2.59 to 3.00) g/dL, 0.98 (95% LoA: − 1.38 to 3.35) g/dL, and 1.23 (95% LoA: − 1.16 to 3.63) g/dL for the normal, mild-to-moderate, and severe groups, respectively. The four-quadrant plot showed reliable trending ability in all groups (concordance rate > 92%). The rates of possible missed transfusion (SpHb > 7.0 g/dL for Hb < 7.0 g/dL) were higher in the hyperbilirubinemia groups (2%, 7%, and 12% for the normal, mild-to-moderate, and severe group, respectively. all P < 0.001). The possible over-transfusion rate was less than 1% in all groups. In conclusion, the use of SpHb in liver transplantation recipients with preoperative hyperbilirubinemia requires caution due to the positive bias and high risk of missed transfusion. However, the reliable trending ability indicated its potential use in clinical settings.

## Introduction

Maintaining adequate hemoglobin (Hb) levels during surgery is crucial for preventing hypoxic organ damage and reducing postoperative complications^1,2^. Although the laboratory measurement of blood Hb concentration is a standard diagnostic method in clinical practice, it is invasive and time-consuming^3^. Alternatively, non-invasive continuous hemoglobin concentration (SpHb) monitors, such as the Radical-7 Pulse CO-Oximeter (Masimo Corp., Irvine, CA), enable continuous non-invasive measurement of Hb level in real-time^4^.

However, previous studies have reported that low or high Hb levels, low perfusion index, fluid infusion, and transfusion can cause inaccuracies in SpHb measurements^5–9^. Additionally, the operator’s manual for the Radical-7 Pulse CO-Oximeter states that elevated serum bilirubin levels may cause erroneous SpHb readings. Transcutaneous spectrophotometry-based techniques, such as SpHb, use multiwavelength absorption properties for estimating Hb concentration. However, the absorption of bilirubin in the wavelength range of 410–500 nm overlaps with the absorption of Hb in the wavelength range of 250–1100 nm^10^. Therefore, bilirubin may be perceived as Hb, resulting in a positive bias in SpHb measurements.

Clinically, inaccuracies in SpHb measurement can lead to under- and over-transfusion. Especially, the Hb concentration of patients with jaundice needs particular attention because these patients are often at risk for preoperative anemia and massive intraoperative bleeding^11,12^. Therefore, it is essential to assess the accuracy of SpHb measurement in patients with jaundice and evaluate the effect of hyperbilirubinemia on the accuracy of the SpHb measurements. However, studies regarding the effect of hyperbilirubinemia on the accuracy of SpHb measurements have been scarce^13^.

Thus, in this study, we aimed to evaluate the accuracy of SpHb measurements from liver transplantation recipients in a prospectively collected cohort registry. We hypothesized that SpHb measurements would be less accurate during hyperbilirubinemia. As a secondary analysis, we assessed the possible over-transfusion (SpHb < 7.0 g/dL for Hb > 7.0 g/dL) and missed transfusion (SpHb > 7.0 g/dL for Hb < 7.0 g/dL) rates based on the SpHb results to evaluate the clinical impact of SpHb measurement inaccuracies.

## Results

### Patient characteristics

**Figure 1:**
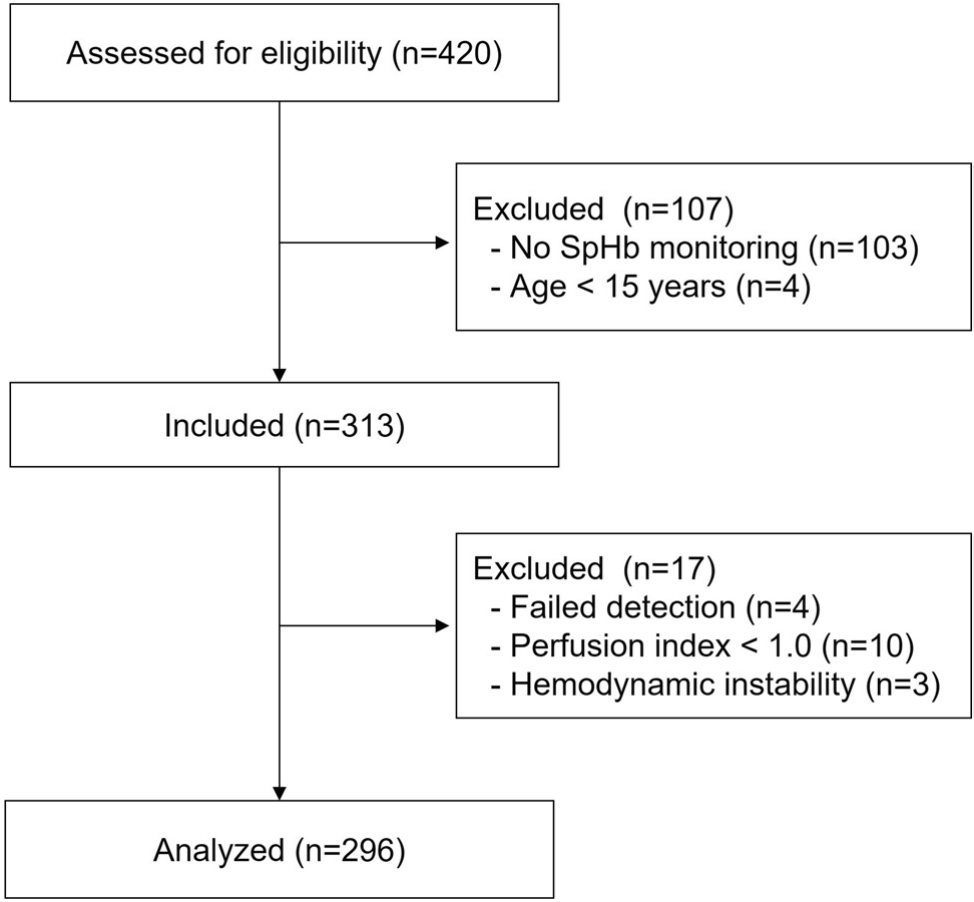
Flow diagram of the present study. SpHb, non-invasive hemoglobin concentration.

Among the 420 patients who underwent liver transplantation, 313 patients who received SpHb monitoring and aged > 15 years were included in this study. After excluding patients with no recorded SpHb measurements, failed detection, hemodynamic instability, or perfusion index < 1.0, we analyzed 1465 simultaneous SpHb, and total Hb measurement pairs from 296 patients (Fig. 1).

Table 1 presents the patient-specific and surgical data for each group. The patients in the severe hyperbilirubinemia group had lower preoperative Hb levels, higher rates of liver transplants from deceased donors, and more intraoperative bleeding. The total bilirubin concentration and MELD scores were significantly different among the groups.

**Table 1:** Patient and surgical characteristics by preoperative serum total bilirubin levels.

	Normal (n = 127)	Mild-to-moderate (n = 87)	Severe (n = 82)	P-value
Age	57 (10)	55 (11)	56 (12)	0.321
Weight (kg)	65 (11)	62 (12)	63 (13)	0.33
MELD scores	8 (3)	12 (3)	23 (9)	< 0.001
Living donor, n (%)	126 (99%)	87 (100%)	61 (74%)	< 0.001
Preoperative hemoglobin (g/dL)	12.4 (2.2)	10.6 (1.8)	9.7 (1.6)	< 0.001
Preoperative total bilirubin (g/dL)	0.7 (0.2)	1.8 (0.5)	13.1 (11.8)	< 0.001
Estimated blood loss (mL)	3151 (4809)	4102 (4360)	7169 (9552)	< 0.001
Surgery duration (min)	431 (136)	432 (127)	419 (139)	0.802

The preoperative serum total bilirubin levels are classified into normal (< 1.2 mg/dL), mild-to-moderate hyperbilirubinemia (1.2–3.0 mg/dL), and severe hyperbilirubinemia (> 3.0 mg/dL ). Values are reported as the mean (standard deviation) or number (percentages).

*SpHb* non-invasive hemoglobin concentration, *MELD* model for end-stage liver disease.

### Accuracy of SpHb measurements

**Figure 2:**
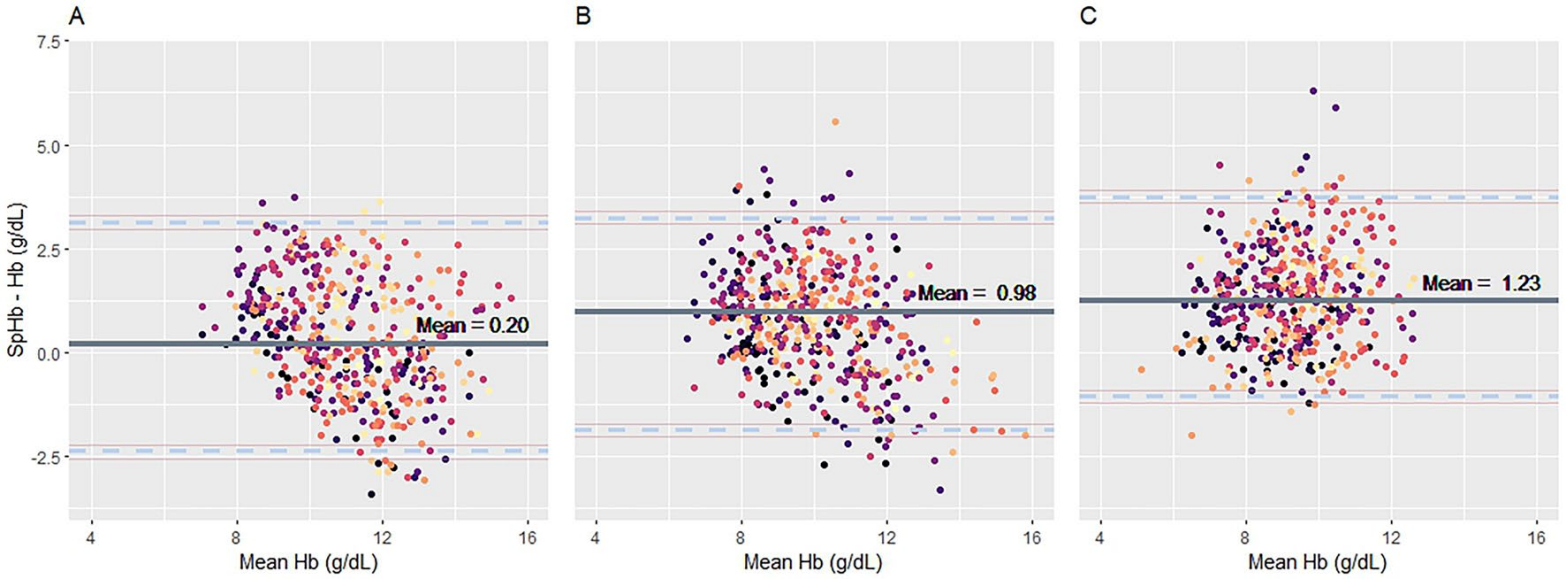
Bland–Altman analysis of hemoglobin measurement error. Bland–Altman plots for repeated measurements per participant comparing SpHb and laboratory hemoglobin concentrations in the normal (**a**), mild-to-moderate hyperbilirubinemia (**b**), and severe hyperbilirubinemia (**c**) groups. The mean bias (solid line) ± 95% confidence interval (dashed line) is displayed. *SpHb* non-invasive hemoglobin concentration.

**Figure 3:**
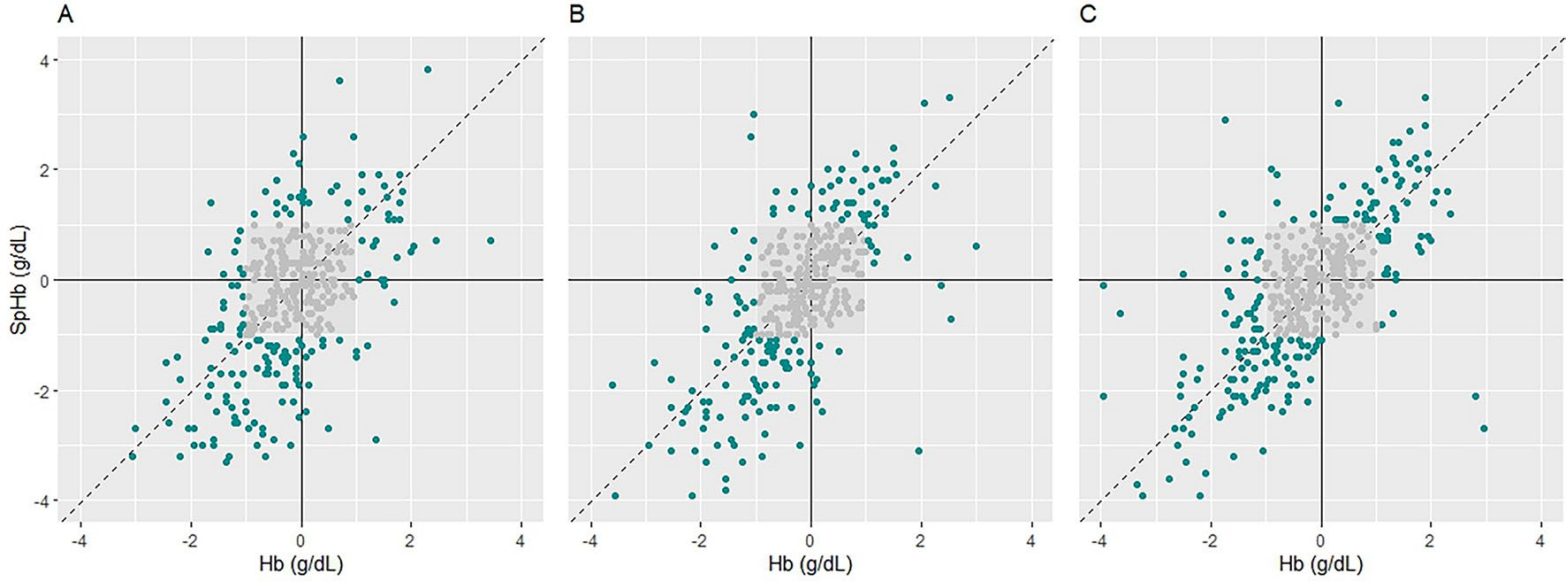
Four-quadrant plots of SpHb and laboratory hemoglobin concentrations. Four-quadrant plots of SpHb and laboratory hemoglobin concentrations in the normal (**a**), mild-to-moderate hyperbilirubinemia (**b**), and severe hyperbilirubinemia (**c**) groups. Data points within the grey box are excluded (hemoglobin 1.0 g/dL). *SpHb* non-invasive hemoglobin concentration.

According to the Bland–Altman analysis, the bias for SpHb and total Hb was 0.20 (95% Limit of agreement, LoA: − 2.59 to 3.00) g/dL with a percentage error of 3%, 0.98 (95% LoA: − 1.38 to 3.35) g/dL with a percentage error of 11%, and 1.23 (95% LoA: − 1.16 to 3.63) g/dL with a percentage error of 20% for the normal, mild-to-moderate, and severe hyperbilirubinemia groups, respectively (P < 0.001) (Fig. 2, Table 2). The trending ability of SpHb was shown in a four-quadrant plot. The concordance rates were 92%, 97%, and 95% in the normal, mild-to-moderate, and severe hyperbilirubinemia groups, respectively, exceeding previously defined reliable criteria (Fig. 3).

**Table 2:** Evaluation of SpHb accuracy among the study groups.

Measures	Normal (n = 127)	Mild-to-moderate (n = 87)	Severe (n = 82)	P-value
Bias (g/dL)	0.20 (− 2.59 to 3.00)	0.98 (− 1.38 to 3.35)	1.23 (− 1.16 to 3.63)	< 0.001
Concordance rate	92%	97%	95%	
Major error rate*	27%	52%	61%	< 0.001
Possible missed transfusion (SpHb > 7.0, Hb < 7.0 g/dL)	2%	8%	12%	< 0.001
Possible over-transfusion (SpHb < 7.0, Hb > 7.0 g/dL)	0.0%	0.2%	0.9%	NA^†^

The preoperative serum total bilirubin levels are classified into normal (< 1.2 mg/dL), mild-to-moderate hyperbilirubinemia (1.2–3.0 mg/dL), and severe hyperbilirubinemia (> 3.0 mg/dL ). Values are reported as the mean (95% limit of agreement), number, or proportion (percentage).

*Proportion of major errors that fall into the grey or red zone in the error grid analysis.

^†^Not available because n > 500, but the number of each cell < 5.

*SpHb* non-invasive hemoglobin concentration, *Hb* hemoglobin.

**Figure 4:**
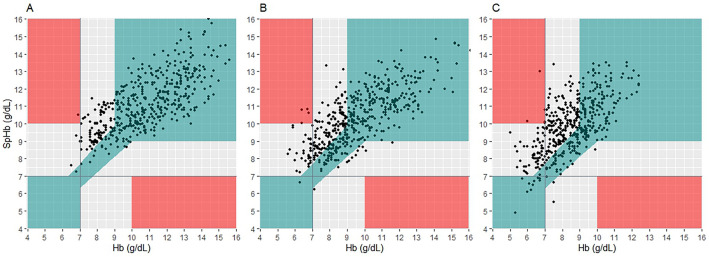
Error grid analysis of SpHb and laboratory hemoglobin concentrations. Error grid analysis of SpHb and laboratory hemoglobin concentrations in the normal (**a**), mild-to-moderate hyperbilirubinemia (**b**), and severe hyperbilirubinemia (**c**) groups show that SpHb overestimated total hemoglobin concentration in severe hyperbilirubinemia patients (P < 0.001). The green zone indicates that the error is within the clinically acceptable range (< 10%) for hemoglobin 7–10 g/dL. The grey zone shows differences greater than 10% with potential major therapeutic error. The red zone represents critical therapeutic error (SpHb < 7 g/dL for Hb > 10 g/dL or SpHb > 10 g/dL for Hb < 10 g/dL). *SpHb* non-invasive hemoglobin concentration.

In the error grid analysis (Fig. 4), the proportion of major errors falling in the grey zone was significantly higher in the mild-to-moderate and severe hyperbilirubinemia groups than in the normal group (frequency: 52% and 61% vs. 27%, P < 0.001) (Table 2). In all groups, there were less than five points (< 1%) that fell within the red zone.

The numbers of possible over- and missed transfusions are presented in Table 2. Possible over-transfusion was rare at < 1% in all groups. However, the possible missed transfusions were more common in the mild-to-moderate and severe hyperbilirubinemia groups than in the normal group (frequency: 8% and 12% vs 2%, P < 0.001).

### Other influencing factors of SpHb accuracy

In the anemia subgroup with Hb < 7.0 g/dL, the SpHb measurement had a consistently higher bias of 1.97 (95% confidence interval, CI: 1.42–2.52), 2.06 (95% CI: 1.67–2.44), and 1.62 (95% CI: 1.32–1.92) for the normal, mild, and severe hyperbilirubinemia groups, respectively. For the subgroup with Hb > 7.0 g/dL, the SpHb measurement had a bias of 0.26 (95% CI: 0.15–0.37), 1.06 (95% CI: 0.94–1.17), and 1.22 (95% CI: 1.09–1.35), respectively.

During massive bleeding, the biases increased to 1.13 (− 0.91 to 3.17), 1.48 (− 1.83 to 4.80), and 1.83 (− 0.51 to 4.17), respectively. Similarly, during blood transfusions, the biases remained elevated: 1.13 (− 1.31 to 3.46), 1.28 (− 1.18 to 3.74), and 1.29 (− 1.01 to 3.58), respectively. The lowest biases were observed in the non-massive bleeding non-transfusion subgroups: 0.20 (− 2.69 to 3.08), 0.69 (− 1.95 to 3.32), and 1.30 (− 1.03 to 3.63), respectively.

The SpHb measurement presented consistent accuracy regardless of whether the perfusion index was above or below the predetermined threshold of 1.4: 0.32 (95% CI: 0.05–0.59), 1.10 (95% CI: 0.85–1.34), and 1.23 (95% CI: 0.97–1.50) for perfusion index < 1.4, respectively and 0.29 (95% CI: 0.18–0.40), 1.14 (95% CI: 1.03–1.26), and 1.28 (95% CI: 1.16–1.40), for perfusion index > 1.4, respectively.

We also investigated the difference between SpHb and Hb measurements according to the surgical stages. The bias of SpHb was 0.73 (95% CI: 0.50–0.96), 0.47 (95% CI: 0.22–0.71), 0.83 (95% CI: 0.61–1.05), and 1.07 (95% CI: 0.84–1.31) for the post-induction, dissection, anhepatic, and neohepatic phases, respectively (P = 0.004). The difference between SpHb and Hb increased with increasing total bilirubin (correlation coefficient (r) = 0.17, P < 0.001) or conjugated bilirubin (r = 0.11, P < 0.001). However, it decreased as the proportion of conjugated bilirubin to total bilirubin increased (r = − 0.15, P < 0.001).

In the univariable analysis, factors such as total Hb, perfusion index, serum total bilirubin, and conjugated bilirubin were all significantly associated with potential major therapeutic errors (all P < 0.001 except for the perfusion index with P = 0.001, Table 3). The subsequent multivariable analysis revealed that decreased total Hb (odds ratio = 0.275 (0.233–0.324), P < 0.001) and increased serum total bilirubin (odds ratio = 1.090 (1.013–1.171), P = 0.020) were independent risk factors of the major therapeutic errors (Table 3).

**Table 3:** Independent risk factors affecting major error* in SpHb measurement.

	Univariable analysis		Multivariable analysis		
	Odds ratios	P-value	Odds ratios	P-value	VIF
Total Hb	0.271 (0.233–0.314)	< 0.001	0.275 (0.233–0.324)	< 0.001	2.1112
Perfusion index	0.900 (0.844–0.956)	< 0.001	1.125 (1.001–1.198)	0.048	1.1247
Serum total bilirubin	1.110 (1.074–1.148)	0.001	1.090 (1.013–1.171)	0.020	3.6576
Conjugated bilirubin	1.165 (1.089–1.247)	< 0.001	0.915 (0.803–1.043)	0.184	2.3009

Values are reported as odds ratio (95% confidence intervals).

*SpHb measurement that falls into the grey or red zone in the error grid analysis.

*SpHb* non-invasive hemoglobin concentration, *Hb* hemoglobin, *VIF* variance inflation factor.

## Discussion

This study evaluated the accuracy and trending ability of SpHb for patients with hyperbilirubinemia who underwent liver transplantation. The severe hyperbilirubinemia group showed a significant increase in the bias and major error rate measurements of SpHb. Nevertheless, the trending ability was reliable irrespective of total bilirubin levels.

Rapid detection and timely treatment of intraoperative anemia are essential in patients with jaundice who are frequently at risk of preoperative anemia, coagulopathy, thrombocytopenia, and platelet dysfunction^11,12,14^. However, the effect of hyperbilirubinemia on the accuracy of SpHb has not been clarified to date. In a previous study, hyperbilirubinemia was suspected of causing SpHb inaccuracy in patients undergoing liver transplantation. However, the findings did not reach statistical significance due to the small sample size^13^.

Our results showed that among patients with severe hyperbilirubinemia, the bias of SpHb rapidly increased to 1.23 (− 1.16 to 3.63) g/dL, and the proportion of major error increased to 61%. Possible missed transfusions were also more frequent in the severe hyperbilirubinemia group, indicating that SpHb as a transfusion trigger can significantly impact clinical decisions^3,7^. Thus, more rigorous methods, such as laboratory tests for measuring Hb concentration, should be utilized in decision making for transfusions to prevent significant complications related to missed transfusion and undertreated anemia (e.g., organ hypoxemia). However, SpHb offers potential clinical benefits when combined with laboratory tests due to its good trending ability, which remains above 95% even in severe hyperbilirubinemia cases. Previous studies have reported good trending ability of SpHb in adult patients undergoing surgery, including liver transplantation^15–18^. Considering the difficulty of frequent blood tests during hemodynamic instability, effective trend-monitoring of SpHb can be clinically valuable. Therefore, we suggest using SpHb trends in clinical situations and calibrating the SpHb monitor frequently by using laboratory hemoglobin measurements, especially in hyperbilirubinemia patients. A previous study has also reported high accuracy of SpHb’s trending ability (R^2^ = 0.96) and concluded that adding SpHb monitoring to the standard of care blood management resulted in decreased blood utilization in high blood loss neurosurgery^17^.

The potential interference between the absorption spectra of hemoglobin and bilirubin, a hemoglobin derivative, could explain the inaccuracy of SpHb measurements in severe hyperbilirubinemia. SpHb monitors transmit light through a body part and measure the absorption of the light. High bilirubin levels can interfere with the readings, especially at lower hemoglobin levels, due to overlapping absorption wavelengths. The wavelength of the light maximally absorbed by hemoglobin is between 400 and 425 nm, which overlaps the light absorption of bilirubin between 400 and 490 nm^19^. Moreover, endogenous hemoglobin derivatives, such as carboxyhemoglobin or methemoglobin, often elevated in severe jaundice patients, could further affect the readings^20^. Therefore, further improvement of the algorithm in the multiwavelength oximetry is required to differentiate between the absorption properties of Hb and bilirubin.

Several factors that can affect SpHb measurements were investigated in this study. Massive bleeding was found to induce inaccuracies, partially improved by transfusion. Hb < 7.0 g/dL showed significant bias, consistent with previous reports^3,6,7^. Preoperative anemia may coincide with hyperbilirubinemia in liver transplant patients, but increased serum total bilirubin independently contributed to major therapeutic errors of SpHb measurements (the grey zone in the error grid plot) in our results. A comparison by surgical stage showed that SpHb was more significantly inaccurate in the neohepatic phase, the most hemodynamically unstable stage after liver reperfusion, than in the dissection phase. Both total bilirubin and conjugated bilirubin caused a positive bias in SpHb, but the bias decreased as the fraction of conjugated bilirubin decreased. This may be due to the adsorption wavelength band of unconjugated bilirubin overlapping more with hemoglobin than conjugated bilirubin. However, further research is needed as these variables are not independent.

Our study had several limitations. Given that our observations were based on uncalibrated SpHb measurements, we cannot draw definitive conclusions regarding the accuracy of the calibrated SpHb. However, calibrating the offset bias using the initial Hb measurement could have enhanced its accuracy. Second, we did not adjust for other confounding factors such as vasopressors^21^, fluid infusion, and hemodynamic instability, which could affect the accuracy of SpHb monitoring^4,7,21,22^. Third, we did not conduct further analyses to provide equations for correcting the effect of bilirubin level on SpHb because correcting the results of the proprietary SpHb algorithm with a single confounding factor may introduce other unpredicted biases.

In conclusion, SpHb monitoring is acceptable for measuring and tracking Hb levels in patients with normal and mild-to-moderate hyperbilirubinemia. However, there are inaccuracies in patients with severe hyperbilirubinemia, except for trending ability. Additionally, SpHb as a transfusion trigger is associated with frequent missed transfusions, and thus, clinical decisions regarding transfusions should be based on laboratory measurements of Hb concentration in patients with severe hyperbilirubinemia.

## Methods

### Study design and ethics

This retrospective cohort study was approved by the Institutional Review Board (IRB) of Seoul National University Hospital (approval no: 2209-100-1359). The IRB of Seoul National University Hospital waived the requirement for informed consent from the participants owing to the retrospective study design. We performed this study in accordance with the Strengthening the Reporting of Observational Studies in Epidemiology (STROBE)^23^, and the Declaration of Helsinki.

### Study population and data collection

This study included patients who received liver transplantation at Seoul National University Hospital between November 2018 and March 2022. Patients without SpHb measurements or those aged < 15 years were excluded. Demographic data, including age, weight, estimated blood loss, the Model for End-stage Liver Disease (MELD) scores^24^, and laboratory test results for total Hb and serum bilirubin levels were obtained from electronic medical records. Estimated blood loss and blood product transfusions were recorded at 5-min intervals.

Time-matched SpHb and perfusion index values were retrieved from the prospectively collected vital signs registry, after approval by the IRB of Seoul National University Hospital (H-1408-101-605), and were registered at a publicly accessible clinical trial registry (ClinicalTrial.gov: NCT02914444).

### Anesthetic management

We implemented our routine anesthesia management protocol for liver transplantation during the study period. Anesthesia was induced with an intravenous bolus of propofol (2.0 mg/kg) and effect-site target-controlled infusion of remifentanil with a concentration range of 3.0–4.0 ng/mL using the Minto model. Tracheal intubation was facilitated with rocuronium (1 mg/kg). After induction of anesthesia, a radial arterial line was inserted using a 20-G catheter to obtain blood samples. Anesthesia was maintained with sevoflurane at 2 vol% to achieve a bispectral index value of 40–60. A Masimo adhesive sensor (R1 20L or R1 25L, rev E) connected to a Radical-7™ Pulse CO-Oximeter, software version 7621 and 7801, was placed on the nail bed of the finger to monitor SpHb continuously. Additionally, a light shield was applied to the fingertip to prevent optical interference.

Routine blood samples were obtained for complete blood count (measuring total Hb) and liver function tests (measuring serum total bilirubin) at eight different time points: immediately after the anesthesia induction, 1 h after induction of anesthesia, 10 min after the beginning of the anhepatic phase, 5 min before and 5 and 20 min after grafted liver reperfusion, 5 min after biliary tract reconstruction, and at the end of the surgery. The exact time of each blood draw was recorded in the anesthesia chart. Total Hb concentration was analyzed using the XE-2100 (Sysmex Corp, Kobe, Japan) and XN (Sysmex Corp) automated hematology systems. Serum total bilirubin level was measured using the TBA 200FR (Toshiba Medical Systems, Ōtawara, Japan) chemistry analyzer.

### Outcome measures

The primary outcome measure was the accuracy of SpHb according to the serum total bilirubin levels. The results were reported as bias, 95% LoA, and percentage error. The secondary outcome measures included the number and rates of possible over-transfusion and missed transfusion based on the SpHb^25^.

### Subgroup analysis

Several factors known to affect the accuracy of SpHb were investigated. Specifically, we conducted further investigations on (1) subgroups with total Hb levels below and above 7.0 g/dL, a threshold that may be a transfusion trigger in critically ill patients^26^; (2) subgroups experiencing massive bleeding, defined as a loss of 150 mL/min^27^, or those receiving a blood transfusion within 5 min before the measurement; (3) subgroups with perfusion index below and above 1.4, ensuring perfusion at a more conservative level in critically ill patients^28^; (4) proportion of conjugated bilirubin to serum total bilirubin; and (5) intraoperative stages, including post-induction, dissection, anhepatic, and reperfusion phases.

### Independent risk factors

We performed the risk factor analysis on major therapeutic errors (the grey or red zones in the error grid plot). The multivariable logistic regression analysis, including factors such as total Hb, perfusion index, serum total bilirubin, and conjugated bilirubin, was conducted following univariable analysis. Odds ratios with 95% CI and the variance inflation factor (VIF) for collinearity diagnosis were calculated.

### Statistical analyses

In calculating the sample size, we made a conservative assumption that hyperbilirubinemia could affect SpHb with an effect size of 0.1. Using G*power Software version 3.1^29^, a minimum sample size of 1367 was needed for a study power of 95% and an alpha error of 0.05. The patients were categorized into three groups based on the last preoperative serum total bilirubin levels: normal (< 1.2 mg/dL), mild-to-moderate hyperbilirubinemia (1.2–3.0 mg/dL), and severe hyperbilirubinemia (> 3.0 mg/dL) groups. The classification adopted the serum total bilirubin criteria of the Child–Pugh scoring system (< 2.0 mg/dL, 1 point; 2.0–3.0 mg/dL, 2 points; > 3.0 mg/dL, 3 points)^30^. The accuracy of SpHb measurement was then compared among the groups.

To ensure the accuracy of the SpHb measurement, any data recorded under a perfusion index of < 1.0 or mean blood pressure of < 60 mmHg was excluded from the final analysis. Data with missing values were also excluded. Descriptive statistics were applied for demographic and clinical data, and the values were reported as the mean ± SD or median (interquartile range). The variables were tested on a measurement rather than a patient basis. Agreement and precision between SpHb and total Hb concentrations in the normal, mild-to-moderate, and severe hyperbilirubinemia groups were evaluated using Bland–Altman analyses with multiple measurements per patient performed using MedCalc Statistical Software version 19.8.0 (MedCalc Software Ltd, Ostend, Belgium)^31^. Repeated measures analysis of variance followed by post-hoc analysis was performed to compare the differences in SpHb and total Hb between groups.

A four-quadrant plot was used to perform a trending ability analysis and compare the differences in consecutive values of SpHb and total Hb to identify the direction and magnitude of changes. A central exclusion zone was applied to exclude clinically insignificant changes < 1.0 g/dL. The concordance rate was calculated as the proportion of the data points falling into the quadrants of agreement (upper right and lower left). Reliable trending ability was defined a priori as a concordance rate > 90%^32,33^.

An error grid analysis was performed to interpret the clinical implications of measurement errors^34^. The error grid plot had three zones based on the practice guidelines for perioperative blood management and clinical decision-making^35–37^. Errors within the green zone had no further therapeutic consequences because the reference and test values had the same impact on decision making. Meanwhile, errors in the grey zone may lead to major therapeutic errors (> 10% in the Hb measurements) in transfusion management, and errors in the red zone (SpHb < 7 g/dL for Hb > 10 g/dL or SpHb > 10 g/dL for Hb < 7 g/dL) may pose a risk to patient safety without possible benefit. The proportions of data in each of the zones were analyzed. Following guidelines that suggest transfusions for Hb levels under 7 g/dL, we calculated missed transfusion rates when SpHb > 7 g/dL with Hb < 7 g/dL, and over-transfusion rates when SpHb < 7 g/dL despite Hb > 7 g/dL^26^. The Chi-square test was performed to compare the number of major therapeutic errors, possible over-transfusion, and possible missed transfusion.

The *nls2* and *ggplot2* R packages were used for non-linear regression analysis and the Bland–Altman plot, respectively. All statistical analyses were conducted using R 3.2.0 statistical software (R Foundation for Statistical Computing, Vienna, Austria). P < 0.05 was considered significant.

## Data Availability

The datasets generated during and/or analyzed during the current study are available from the corresponding author on reasonable request.
